# Stillbirths in Germany: On the rise, but no additional increases during the first COVID‐19 lockdown

**DOI:** 10.1002/ijgo.13832

**Published:** 2021-08-07

**Authors:** Maxi S. Kniffka, Natalie Nitsche, Roland Rau, Mine Kühn

**Affiliations:** ^1^ Laboratory of Fertility and Well‐Being Max Planck Institute for Demographic Research Rostock Germany; ^2^ Department of Sociology and Demography University of Rostock Rostock Germany; ^3^ Department of Sociology and Demography Chair of Demography University of Rostock Rostock Germany; ^4^ Laboratory of Statistical Demography Max Planck Institute for Demographic Research Rostock Germany; ^5^ Laboratory of Population Health Max Planck Institute for Demographic Research Rostock Germany

**Keywords:** COVID‐19, fetal death, Germany, lockdown, stillbirth

## Abstract

**Objective:**

To examine possible changes in the rate of stillbirths in Germany during the first COVID‐19 lockdown.

**Methods:**

Population‐level data of live births and stillbirths occurring between January 1995 and July 2020 were used and negative binomial regression was applied to estimate the rate of stillbirths in this period. The actual rate was compared to the expected figure for 2020.

**Results:**

A steady increase in stillbirths was detected in Germany since 2013. The stillbirth rate for January to July 2020 (4.148) was slightly lower than that of the same period in 2019 (4.242). Furthermore, all monthly rates of stillbirths during the first half of 2020 lie inside the 95% prediction interval of expected stillbirth rates for this period.

**Conclusion:**

A growing body of studies on the indirect effect of the COVID‐19 pandemic on stillbirths shows mixed and context‐dependent evidence. In contrast to other European countries, stillbirth rates have been on the rise in Germany in the last decade. However, stillbirth rates during the first half of 2020 were not higher than expected. The results suggest that stillbirth rates have not changed during the first‐wave COVID‐19 lockdown in this high‐income setting. However, further studies on the causes of the increasing trend in stillbirths in Germany are needed.

## Synopsis

The rate of stillbirths has been increasing in Germany over the last decade but was not higher than expected during the first wave of COVID‐19.

## 1 INTRODUCTION

As in many other countries affected by the COVID‐19 pandemic, the German federal and state governments responded to increasing numbers of cases of COVID‐19 and deaths by taking drastic public health measures intended to slow down the rate of transmission of the SARS‐CoV‐2 virus. During the pandemic's first wave, large parts of public life were shut down from March 22, 2020, for the next 3 months. To keep intensive care beds available, the German government ordered hospitals to postpone non‐emergency procedures and surgeries. First comparisons of hospitalizations during the pandemic (March 16 to April 5, 2020) and pre‐pandemic (corresponding dates in 2019) periods, based on data from one of the largest insurance companies in Germany, indicate a decrease of 16% in pregnancies, births, or postpartum‐related hospitalizations and a decrease of 14% in hospitalizations related to diseases of the fetus or the newborn.^1^

Scholars expressed concerns from the early stages of the pandemic that the COVID‐19 measures might disrupt the delivery of health services, including reproductive and maternal healthcare services, and would reduce care‐seeking behavior. The scientific community consequently called for studies on the indirect effect of COVID‐19 on perinatal and maternal outcomes in general and on stillbirths in particular.^2^

One year later, evidence from the growing body of studies investigating the possible correlation between COVID‐19 lockdown measures and stillbirths reflects mixed results. A recently published meta‐analysis indicates that the rates of stillbirths have increased during the pandemic, particularly in low‐ and middle‐income countries, but not in high‐income settings.^3^ Indeed, several hospital‐based studies from low‐, middle‐, and high‐income countries have reported a rise in the rate of stillbirths during the pandemic. There is evidence that the risk of stillbirths significantly increased in some hospitals in Nepal and India during the lockdown in the spring of 2020 compared to the weeks before the lockdown.^4^, ^5^ Similarly, a study comparing the incidence of stillbirths based on data from one hospital in London during the pandemic (February to June 2020) to the pre‐pandemic period (October 2019 to January 2020), found an increase in the incidence of stillbirths from 2.38 per 1000 births to 9.31 per 1000 births, none of which were associated with known COVID‐19 infections.^6^ A study based on one hospital in Israel indicated a significant increase in the rates of stillbirths in February to April 2020, compared to the corresponding periods in 2017 to 2019.^7^ Another study, based on a database of hospital discharges from the Lazio region in Italy, reported an increase in stillbirth rates when comparing the period of March to May 2020 with the corresponding period in 2019.^8^

In contrast, no evidence for an increase was found in a study based on data from two Philadelphia (USA) hospitals that compared rates of stillbirths for the period of March to June 2020 with the corresponding periods in 2018 and 2019.^9^ This finding is in line with the four available studies on stillbirths in high‐income countries based on population‐level data. There was no evidence of increases in stillbirths in England, regionally or nationally, in the period of April to June 2020 compared to the same period in 2019,^10^ or when a longer pre‐pandemic period of up to 5 years was taken into account in studies on the Castilla y León region in Spain,^11^ Sweden,^12^ and Austria.^13^

These mixed results on stillbirths in high‐income countries may be caused by issues of selectivity in the studies using hospital data; findings from the representative population‐level studies are likely more robust. Nonetheless, most previous studies that explored the indirect effect of the COVID‐19 pandemic on stillbirths focused on comparing rates of stillbirth during the pandemic to relatively short pre‐pandemic periods, in the range of 1–5 years before the pandemic. To account for random fluctuations in the number of stillbirth events and for pre‐pandemic trends in the rates of stillbirths, particularly in high‐income countries where stillbirths are rare, it is, however, important to consider longer‐term trends of stillbirths when assessing potential changes during the pandemic. The rates of stillbirths in most high‐income countries have continually declined in the last few decades, despite differences in overall stillbirth burdens and the magnitude of decline.^14^, ^15^ This means that assessments of pandemic‐related changes in stillbirths in 2020 would ideally be based on comparing the observed numbers of stillbirths during a specific 2020 pandemic time window with the expected number of stillbirths for the same period. Therefore, the aim of the present study was to assess the indirect effect of the COVID‐19 pandemic on stillbirths in Germany by comparing the observed rate of stillbirths in January to July 2020 with the expected rate considering the long‐term trends in stillbirths since 1995, using full population data from statistical offices.

## 2 MATERIALS AND METHODS

The data for pre‐pandemic rates of stillbirths in Germany was gathered from the Federal Statistical Office.^16^, ^17^ They provided the total number of live and stillbirth events in Germany on a monthly basis from 1990 up to 2019, as well as the monthly number of live births for each federal state. The monthly provisional numbers of births for the whole of Germany and for each federal state from January to July 2020 were also provided by the Federal Statistical Office. Since the official statistics for stillbirths are not compiled until the summer of the following year, the provisional figures for stillbirths had to be obtained from the statistical offices of the respective federal states.^18^, ^19^, ^20^, ^21^, ^22^, ^23^, ^24^, ^25^, ^26^, ^27^, ^28^, ^29^, ^30^, ^31^, ^32^ With the exception of Thuringia, the number of stillbirths from January to July 2020 were provided by the statistical offices of each federal state. All numbers for 2020 are preliminary figures. Since the statistical office of Thuringia did not provide stillbirth data for 2020, data were collected on the monthly number of stillbirths from 1995 until 2019 for Thuringia to enable the exclusion of Thuringia's live births and stillbirths from the present analysis.^33^, ^34^, ^35^, ^36^, ^37^, ^38^, ^39^, ^40^, ^41^, ^42^, ^43^, ^44^, ^45^, ^46^, ^47^, ^48^, ^49^, ^50^

The observed monthly rate of stillbirths in January through July 2020 was then compared with the following: (1) the observed rate of stillbirths in 2019; and (2) the predicted number of stillbirths for the same time period. The prediction is based on the trend in stillbirths detected in the data from January 1995 through December 2019. The prediction rests on a Generalized Additive Model (GAM),^51^, ^52^ where it is assumed that the number of stillbirths follows a negative binomial distribution.^53^, ^54^ The total number of births, that is, stillbirths and live births combined, serves as a (log) offset. This offset does not only take care of changes in the underlying population structure, but also eliminates the effect of different lengths of months. The GAM contains two smooth components: one for the secular trend of stillbirths observed over time, and one for the seasonal component, to adjust for seasonality in both the occurrence of births and stillbirths. Both smooth components are estimated using P‐splines,^55^ which are widely used to estimate, smooth, and analyze mortality.^56^, ^57^ The definition of what counts as a stillbirth has changed twice in Germany during the observation period.^17^ Therefore, an indicator variable measures whether the old definition (January 1995 to October 2018) or the new definition (November 2018 to date) of stillbirths was in use. Prediction intervals were derived analytically using the quantile function of the negative binomial distribution with the seven estimates for the mean (μ1, μ2, μ3, …, μ7) from the GAM for January, February, March,..., July 2020 and the estimate for parameter θ, which controls the level of overdispersion. All statistical analyses were performed using R (R Foundation for Statistical Computing, Vienna, Austria). Ethical approval from an institutional review board and informed consent from any participants were not needed, because the data were de‐identified and (soon to be) publicly available data on the occurrence of stillbirths from statistical offices. Such data do not constitute human subject research.

## 3 RESULTS

Table 1 shows a descriptive comparison between the observed rate of stillbirths for the periods January to July 2019 and 2020, respectively. A slight decrease in the observed rate of stillbirths is indicated in the time window of January to July 2019 (4.242) when compared with the same time window in 2020 (4.148). The descriptive stillbirth rate for 2020 was compared with the rate for 2019 because the potential effect of the definition change for 2018 in the descriptive results could not be controlled for.

**TABLE 1:** Monthly observed and predicted rates of stillbirths with seasonality for Germany, except Thuringia, 2019 to 2020^a^

Year	Month	Observed rate of stillbirths	Predicted rate of stillbirths (95% CI)
2020	Jan	3.694	4.248 (3.677–4.846)
2020	Feb	3.901	4.113 (3.551–4.706)
2020	Mar	4.674	4.170 (3.607–4.758)
2020	Apr	3.981	4.265 (3.694–4.858)
2020	May	4.617	4.290 (3.735–4.869)
2020	Jun	4.158	4.207 (3.658–4.781)
2020	Jul	3.993	3.966 (3.443–4.499)
2019	Mean Jan to Jul	4.242	…
2020	Mean Jan to Jul	4.148	…

^a^ Rates were predicted by a negative binomial model in a Generalized Additive Model framework controlling for definition change and seasonality, estimated “overdispersion‐theta” being 1029.215.^16^, ^17^, ^18^, ^19^, ^20^, ^21^, ^22^, ^23^, ^24^, ^25^, ^26^, ^27^, ^28^, ^29^, ^30^, ^31^, ^32^, ^33^, ^34^, ^35^, ^36^, ^37^, ^38^, ^39^, ^40^, ^41^, ^42^, ^43^, ^44^, ^45^, ^46^, ^47^, ^48^, ^49^, ^50^

**FIGURE 1:**
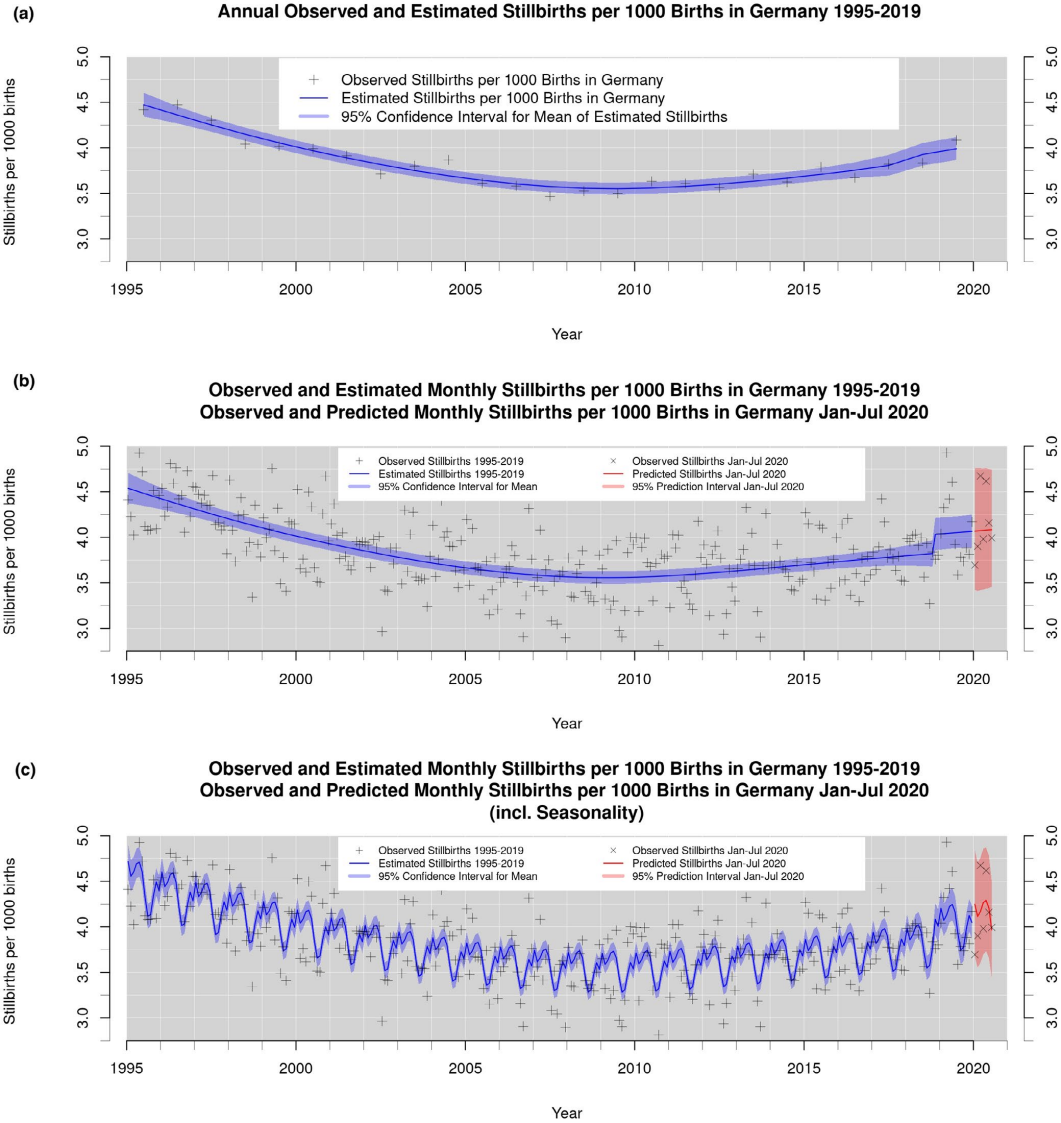
(a) Annually observed and estimated rates of stillbirths for Germany, except Thuringia, from 1995 until 2019, estimated by a negative binomial model in a Generalized Additive Model framework controlling for definition change; (b) monthly observed, estimated, and predicted rates of stillbirths for Germany, except Thuringia, from 1995 until 2020, estimated and predicted by a negative binomial model in a Generalized Additive Model framework controlling for definition change; (c) monthly observed, estimated, and predicted rates of stillbirths for Germany, except Thuringia, from 1995 until 2019, estimated by a negative binomial model in a Generalized Additive Model framework controlling for definition change and seasonality^16^, ^17^, ^18^, ^19^, ^20^, ^21^, ^22^, ^23^, ^24^, ^25^, ^26^, ^27^, ^28^, ^29^, ^30^, ^31^, ^32^, ^33^, ^34^, ^35^, ^36^, ^37^, ^38^, ^39^, ^40^, ^41^, ^42^, ^43^, ^44^, ^45^, ^46^, ^47^, ^48^, ^49^, ^50^, ^51^, ^52^, ^53^, ^54^, ^55^, ^56^, ^57^, ^58^, ^59^, ^60^, ^61^, ^62^

Figure 1 displays the rates of stillbirths in Germany from January 1995 until July 2020. Figure 1a shows annual observed and estimated stillbirth rates, with 95% confidence intervals around the estimated means. The rate of stillbirths in Germany continuously decreased between 1995 and 2006, but then plateaued until 2012. However, a reversal of this trend occurred in 2013; an ongoing increase in the rate of stillbirths has occurred since.

Between 2012 and 2018, the rate of stillbirths in Germany increased by 7.608%. Figures 1b,c displays the monthly rate of stillbirths from January 1995 until July 2020. The estimates in Figure 1c reflect the control for seasonality in the rates of live births and stillbirths, whereas the estimates in Figure 1b do not. The monthly rates of stillbirths from January 2020 to July 2020 lie in the 95% prediction interval for both types of predictions. Hence, the rate of stillbirths in Germany in the first half of 2020 has been neither higher nor lower than the expected rate without the impact of the pandemic. Note that predicted rates of stillbirths for 2020 are slightly higher than estimates in previous years, given the increasing trend over time in German stillbirths.

Table 1 displays monthly predicted rates of stillbirths for 2020. Tables S1 and S2 display the annual and monthly estimations of stillbirths until 2019.

## 4 DISCUSSION

The present study examines the rates of stillbirths in Germany before and during the first wave of the COVID‐19 pandemic. To assess whether the pandemic has indirectly affected the occurrence of stillbirths, observed rates of stillbirths from January 2020 to July 2020 were compared with the observed rates of stillbirths in 2019 and the predicted rates of stillbirths for the first half of 2020, which were derived from modeling trends of stillbirths since 1995. The analyses used full population data (except for the state of Thuringia) obtained from German statistical offices. It was discovered that rates of stillbirths neither increased nor decreased during the first wave of the COVID‐19 pandemic. The observed rate of stillbirths from January to July 2020 was slightly lower than in the corresponding period of 2019. All monthly rates of stillbirths observed in the first half of 2020 lie within the 95% confidence interval of expected rates for this period.

The present results corroborate the findings from the few other available studies on high‐income countries based on population‐level data, which have detected no increase in stillbirths during the first wave of the COVID‐19 pandemic.^9^, ^10^, ^11^, ^12^, ^13^ Furthermore, the findings of the present study show the importance of accounting for underlying trends in the rates of stillbirths when assessing potential pandemic‐related changes. An increasing and previously undocumented population‐level trend was detected in the stillbirth rate in Germany from 2013 onward. In these analyses, the findings on stability in stillbirth rates during the first pandemic wave are robust, regardless of whether mean rates from 2020 were compared to those in 2019, or monthly observed rates of stillbirths were compared with expected monthly rates based on estimates using long‐term trends in stillbirth rates. However, declining (or increasing) trends in the rates of stillbirths that may have been present before the pandemic in other countries or regions should be factored in when assessing indirect effects of the pandemic on the rates of stillbirths and other perinatal outcomes. An increase in the rates of late fetal death from 2008 to 2012 in Germany was also reported by Schwarz et al. ^58^, based on routine perinatal hospital data covering 98.5% of births in Germany. They found a correlation between an overall increase in maternal age and neonatal morbidity.^58^ Further research on the causes of the ongoing increasing trend in stillbirths in Germany is needed. It should, for instance, examine whether the increase in the rate of stillbirths after 2012 is related to increases in maternal age, and whether it may be caused by increasing proportions of pregnancies conceived with the help of assisted reproduction technology, which have a slightly elevated risk of stillbirths.^59^

The major strength of the present study lies in the full‐coverage population‐level data. The data reflect the total population of Germany instead of selected hospitals only. Furthermore, the extensive data coverage of monthly rates from 1995 to 2020 made it possible to analyze the indirect effects of the pandemic on stillbirths in the context of the long‐term trend in Germany, which revealed a previously undocumented ongoing increase in the country’s rate of stillbirths since 2013. The monthly estimates also made it possible to correct for seasonality in expected rates of live births and stillbirths.

Despite its strengths, the present study has some limitations. The aggregated level data do not provide information that allows for analyses of the distribution or causes of the stillbirths. Hence, no detailed information on the mothers or pregnancies, such as age, week of pregnancy, or maternal illnesses, is available. Further, only the indirect effect of the pandemic and the lockdown can be measured because no information on the incidence of cases of COVID‐19 is available in the present data. Women with COVID‐19 infection may carry a double burden; studies show that they run an increased risk of stillbirth, which could be due to either the direct effect of an infection or the indirect effect of the pandemic.^60^ Further, the data are provisional, and may not be 100% accurate. The preliminary data for live births and stillbirths in 2020 reflect the month they were reported and not the month they occurred. Thus, the monthly distribution of stillbirths in the final data, which will be published in the summer of 2021, may vary slightly from the provisional data that are currently used. However, it is unlikely that the total number of stillbirths in the first half of 2020 will change, and the results of the present study should therefore not be affected.^21^ Another limitation is the restricted comparability between the years before and after Germany’s change of the definition of stillbirth in November 2018. Even though allowances were made for the change of definition, it should be mentioned that it is best to compare the data from 2020 with the data from 2019.

No changes were documented in the rates of stillbirths during the first wave of the COVID‐19 pandemic in Germany, but it should be noted that the present study only covers the period until the end of July 2020. In Germany, the rate of total documented cases of COVID‐19 infection during the first wave was low, while the number of infections during the second and subsequent waves (which started after the observation window period in the present study) was higher, and the healthcare system was more affected.^61^ It will be important to analyze the potential changes in the rate of stillbirths when more data become available in the summer of 2021.

Finally, the findings of the present study are context dependent. Germany has a high‐quality healthcare system that provides extensive medical services to almost all pregnant women.^62^

Furthermore, in April 2020, the German Board and College of Gynecology and Obstetrics appealed to pregnant woman to go to hospital when needed and to not decline necessary clinic visits, which could result in poor pregnancy outcomes such as stillbirths.^63^ These factors may have contributed to the stability of the rates of stillbirths during the first wave of the COVID‐19 pandemic in Germany.

## CONFLICTS OF INTEREST

The authors have no conflicts of interest.

## AUTHOR CONTRIBUTIONS

MSK, NN, and MK conceptualized the study. MSK collected and prepared the data. MSK, NN, and MK verified the data. RR performed the data analysis and visualized the results. MSK wrote the first draft of the manuscript. All authors contributed to the subsequent drafts and to the final manuscript. All authors had full access to all the data in the study and all authors had the final responsibility for the decision to submit it for publication.

## Supporting information

Supplementary Material
