# Supplementary material for: Stillbirths in Germany: On the rise, but no additional increases during the first COVID‐19 lockdown
Source: Int J Gynaecol Obstet. 2021 Aug 7;155(3):483–9. doi: 10.1002/ijgo.13832 (PMC9087793; doi:10.1002/ijgo.13832)
Supplement: Supplementary file 1 — Supplementary Material [file IJGO-155-483-s001.pdf]

Supporting Information

Year	Observed stillbirth rate	Estimated stillbirth rate (95 % CI)
1995	4.417	4.473 4.344 4.605
1996	4.473	4.360 4.268 4.454
1997	4.303	4.251 4.176 4.328
1998	4.041	4.149 4.077 4.222
1999	4.018	4.054 3.984 4.126
2000	3.990	3.968 3.900 4.037
2001	3.914	3.889 3.821 3.958
2002	3.711	3.816 3.749 3.885
2003	3.801	3.751 3.686 3.817
2004	3.867	3.693 3.629 3.758
2005	3.611	3.643 3.578 3.710
2006	3.580	3.603 3.539 3.669
2007	3.467	3.574 3.511 3.638
2008	3.525	3.556 3.492 3.622
2009	3.499	3.551 3.487 3.617
2010	3.631	3.559 3.495 3.624
2011	3.608	3.577 3.512 3.643
2012	3.562	3.602 3.535 3.670
2013	3.710	3.632 3.566 3.699
2014	3.624	3.668 3.602 3.734
2015	3.790	3.708 3.638 3.780
2016	3.673	3.755 3.671 3.840
2017	3.821	3.807 3.694 3.923
2018	3.833	3.927 3.805 4.052
2019	4.085	3.989 3.866 4.117

Table S1: Annually observed and estimated stillbirth rates for Germany except Thuringia, from 1995 until 2019, estimated by negative binomial model in a Generalized Additive Model framework controlling for definition change, estimated 'overdispersion-theta' is 7125.652 [16,17,33-50]

Year	Month	Observed stillbirth rate	Estimated stillbirth rate (95% CI)
1995	Jan	4.411	4.718 4.530 4.913
1995	Feb	4.226	4.555 4.387 4.728
1995	Mar	4.024	4.603 4.443 4.770
1995	Apr	5.496	4.694 4.534 4.860
1995	May	4.926	4.709 4.550 4.873
1995	Jun	4.720	4.603 4.449 4.763
1995	Jul	4.120	4.327 4.184 4.474
1995	Aug	4.077	4.116 3.983 4.253
1995	Sep	4.083	4.137 4.006 4.271
1995	Oct	4.515	4.344 4.208 4.484
1995	Nov	4.093	4.493 4.348 4.642
1995	Dec	4.461	4.395 4.239 4.556
1996	Jan	4.538	4.600 4.446 4.759
1996	Feb	4.233	4.441 4.307 4.579

1996	Mar	4.331	4.489	4.361	4.620
1996	Apr	4.807	4.577	4.450	4.708
1996	May	4.591	4.591	4.464	4.722
1996	Jun	4.764	4.489	4.363	4.618
1996	Jul	4.422	4.219	4.102	4.340
1996	Aug	4.461	4.013	3.904	4.126
1996	Sep	4.057	4.034	3.926	4.145
1996	Oct	4.728	4.236	4.122	4.353
1996	Nov	4.225	4.381	4.257	4.509
1996	Dec	4.554	4.286	4.148	4.428
1997	Jan	4.549	4.486	4.349	4.628
1997	Feb	4.472	4.331	4.213	4.453
1997	Mar	4.377	4.378	4.266	4.493
1997	Apr	4.463	4.464	4.352	4.580
1997	May	4.351	4.478	4.365	4.595
1997	Jun	4.353	4.378	4.265	4.495
1997	Jul	4.678	4.116	4.009	4.225
1997	Aug	4.138	3.915	3.815	4.018
1997	Sep	4.159	3.935	3.836	4.037
1997	Oct	4.082	4.133	4.028	4.241
1997	Nov	4.125	4.275	4.159	4.394
1997	Dec	3.820	4.182	4.051	4.317
1998	Jan	4.629	4.378	4.246	4.514
1998	Feb	4.078	4.227	4.113	4.344
1998	Mar	3.734	4.273	4.165	4.383
1998	Apr	4.236	4.358	4.250	4.469
1998	May	4.434	4.372	4.262	4.484
1998	Jun	4.248	4.274	4.165	4.387
1998	Jul	3.848	4.018	3.915	4.124
1998	Aug	3.945	3.823	3.726	3.922
1998	Sep	3.342	3.843	3.747	3.942
1998	Oct	4.351	4.037	3.934	4.142
1998	Nov	3.924	4.175	4.062	4.291
1998	Dec	3.785	4.085	3.958	4.217
1999	Jan	4.212	4.277	4.148	4.410
1999	Feb	3.994	4.130	4.018	4.245
1999	Mar	4.089	4.175	4.070	4.283
1999	Apr	4.756	4.258	4.153	4.367
1999	May	3.854	4.273	4.165	4.383
1999	Jun	4.319	4.178	4.071	4.288
1999	Jul	3.490	3.928	3.828	4.031
1999	Aug	3.779	3.737	3.643	3.834
1999	Sep	3.410	3.758	3.664	3.853
1999	Oct	4.169	3.947	3.848	4.049
1999	Nov	4.146	4.083	3.973	4.196
1999	Dec	4.146	3.996	3.871	4.124
2000	Jan	4.525	4.184	4.057	4.314
2000	Feb	3.654	4.04	3.931	4.152
2000	Mar	3.760	4.085	3.982	4.19

2000	Apr	3.617	4.167	4.064	4.273
2000	May	4.497	4.181	4.076	4.289
2000	Jun	4.335	4.089	3.984	4.196
2000	Jul	3.667	3.845	3.747	3.946
2000	Aug	3.514	3.659	3.566	3.753
2000	Sep	3.502	3.679	3.587	3.773
2000	Oct	4.257	3.865	3.767	3.965
2000	Nov	4.671	3.998	3.891	4.109
2000	Dec	3.961	3.913	3.791	4.039
2001	Jan	4.147	4.098	3.973	4.226
2001	Feb	4.367	3.958	3.850	4.068
2001	Mar	4.022	4.002	3.900	4.106
2001	Apr	3.765	4.082	3.980	4.187
2001	May	4.049	4.097	3.993	4.203
2001	Jun	3.790	4.007	3.903	4.113
2001	Jul	3.693	3.768	3.671	3.868
2001	Aug	3.642	3.586	3.494	3.680
2001	Sep	3.735	3.606	3.515	3.699
2001	Oct	3.954	3.789	3.692	3.888
2001	Nov	3.925	3.920	3.814	4.030
2001	Dec	3.951	3.837	3.716	3.961
2002	Jan	3.946	4.018	3.895	4.145
2002	Feb	3.915	3.881	3.775	3.990
2002	Mar	3.670	3.925	3.825	4.028
2002	Apr	3.979	4.004	3.904	4.108
2002	May	3.885	4.019	3.917	4.124
2002	Jun	4.007	3.931	3.829	4.036
2002	Jul	2.964	3.697	3.602	3.795
2002	Aug	3.420	3.519	3.429	3.611
2002	Sep	3.409	3.539	3.450	3.630
2002	Oct	3.882	3.719	3.624	3.816
2002	Nov	3.708	3.848	3.744	3.955
2002	Dec	3.883	3.766	3.649	3.888
2003	Jan	4.672	3.945	3.825	4.069
2003	Feb	3.489	3.811	3.707	3.917
2003	Mar	4.297	3.854	3.757	3.955
2003	Apr	3.624	3.933	3.835	4.034
2003	May	4.032	3.948	3.848	4.050
2003	Jun	4.277	3.862	3.763	3.964
2003	Jul	3.564	3.633	3.539	3.728
2003	Aug	3.525	3.458	3.370	3.548
2003	Sep	3.549	3.478	3.391	3.567
2003	Oct	3.541	3.655	3.563	3.750
2003	Nov	3.238	3.783	3.681	3.888
2003	Dec	3.792	3.703	3.588	3.822
2004	Jan	4.130	3.879	3.762	4.001
2004	Feb	3.569	3.748	3.646	3.853
2004	Mar	3.758	3.791	3.695	3.890
2004	Apr	4.402	3.869	3.773	3.968

2004	May	4.004	3.884	3.786	3.985
2004	Jun	4.059	3.801	3.703	3.901
2004	Jul	3.902	3.576	3.483	3.670
2004	Aug	3.898	3.404	3.317	3.493
2004	Sep	3.634	3.424	3.338	3.513
2004	Oct	3.449	3.599	3.508	3.694
2004	Nov	3.966	3.726	3.625	3.830
2004	Dec	3.640	3.648	3.534	3.766
2005	Jan	4.196	3.823	3.706	3.943
2005	Feb	3.476	3.694	3.593	3.798
2005	Mar	4.096	3.737	3.641	3.836
2005	Apr	3.670	3.815	3.718	3.914
2005	May	3.663	3.830	3.732	3.931
2005	Jun	3.636	3.749	3.651	3.849
2005	Jul	3.321	3.527	3.435	3.622
2005	Aug	3.740	3.359	3.272	3.448
2005	Sep	3.151	3.380	3.294	3.468
2005	Oct	3.422	3.553	3.462	3.647
2005	Nov	3.775	3.679	3.578	3.783
2005	Dec	3.211	3.603	3.489	3.720
2006	Jan	3.507	3.776	3.660	3.895
2006	Feb	3.559	3.650	3.549	3.753
2006	Mar	3.650	3.693	3.598	3.791
2006	Apr	3.680	3.771	3.675	3.869
2006	May	3.907	3.787	3.690	3.886
2006	Jun	3.797	3.707	3.610	3.806
2006	Jul	3.505	3.489	3.398	3.582
2006	Aug	3.158	3.323	3.238	3.411
2006	Sep	2.906	3.345	3.260	3.431
2006	Oct	3.358	3.517	3.427	3.610
2006	Nov	4.315	3.643	3.543	3.745
2006	Dec	3.760	3.568	3.456	3.684
2007	Jan	3.460	3.741	3.626	3.858
2007	Feb	3.558	3.616	3.518	3.718
2007	Mar	4.080	3.661	3.567	3.756
2007	Apr	3.415	3.738	3.644	3.835
2007	May	3.645	3.756	3.660	3.853
2007	Jun	3.752	3.677	3.582	3.775
2007	Jul	3.083	3.462	3.372	3.554
2007	Aug	3.513	3.298	3.214	3.385
2007	Sep	3.045	3.320	3.237	3.406
2007	Oct	3.587	3.493	3.403	3.584
2007	Nov	3.626	3.618	3.52	3.720
2007	Dec	2.897	3.545	3.434	3.660
2008	Jan	3.773	3.718	3.604	3.835
2008	Feb	3.351	3.595	3.497	3.697
2008	Mar	3.340	3.640	3.547	3.736
2008	Apr	3.602	3.719	3.625	3.815
2008	May	3.703	3.737	3.641	3.835

2008	Jun	3.853	3.660	3.564	3.758
2008	Jul	3.407	3.447	3.357	3.539
2008	Aug	3.324	3.285	3.200	3.372
2008	Sep	3.361	3.308	3.223	3.395
2008	Oct	3.296	3.481	3.391	3.573
2008	Nov	3.502	3.607	3.507	3.709
2008	Dec	3.804	3.535	3.423	3.651
2009	Jan	3.651	3.708	3.594	3.826
2009	Feb	3.322	3.587	3.488	3.689
2009	Mar	3.868	3.633	3.539	3.730
2009	Apr	3.211	3.713	3.618	3.810
2009	May	3.300	3.732	3.636	3.831
2009	Jun	3.962	3.657	3.560	3.755
2009	Jul	2.974	3.445	3.354	3.538
2009	Aug	2.904	3.284	3.198	3.371
2009	Sep	3.195	3.308	3.223	3.395
2009	Oct	3.830	3.482	3.392	3.575
2009	Nov	4.000	3.609	3.510	3.711
2009	Dec	3.921	3.539	3.427	3.654
2010	Jan	3.994	3.713	3.599	3.831
2010	Feb	3.189	3.593	3.494	3.695
2010	Mar	3.891	3.640	3.546	3.737
2010	Apr	3.99	3.721	3.627	3.818
2010	May	3.721	3.742	3.646	3.840
2010	Jun	3.681	3.667	3.571	3.765
2010	Jul	3.303	3.455	3.365	3.548
2010	Aug	3.713	3.295	3.210	3.382
2010	Sep	2.814	3.320	3.235	3.407
2010	Oct	3.808	3.495	3.405	3.588
2010	Nov	3.768	3.624	3.525	3.726
2010	Dec	3.792	3.554	3.442	3.670
2011	Jan	3.227	3.730	3.616	3.848
2011	Feb	4.025	3.611	3.512	3.712
2011	Mar	3.882	3.659	3.565	3.755
2011	Apr	4.023	3.741	3.646	3.838
2011	May	3.452	3.762	3.666	3.861
2011	Jun	3.522	3.688	3.592	3.787
2011	Jul	3.383	3.476	3.385	3.569
2011	Aug	3.268	3.315	3.229	3.403
2011	Sep	3.206	3.341	3.256	3.429
2011	Oct	3.583	3.518	3.427	3.612
2011	Nov	4.175	3.648	3.548	3.752
2011	Dec	3.778	3.579	3.466	3.696
2012	Jan	3.917	3.757	3.641	3.876
2012	Feb	3.706	3.637	3.536	3.740
2012	Mar	3.141	3.686	3.590	3.784
2012	Apr	3.810	3.769	3.672	3.868
2012	May	3.902	3.791	3.693	3.892
2012	Jun	4.071	3.717	3.618	3.818

2012	Jul	3.770	3.503	3.411	3.599
2012	Aug	2.936	3.342	3.254	3.432
2012	Sep	3.161	3.368	3.281	3.458
2012	Oct	3.654	3.547	3.454	3.643
2012	Nov	3.454	3.679	3.577	3.784
2012	Dec	3.301	3.609	3.494	3.728
2013	Jan	3.658	3.789	3.672	3.909
2013	Feb	3.759	3.668	3.566	3.773
2013	Mar	3.865	3.718	3.621	3.817
2013	Apr	4.323	3.802	3.705	3.902
2013	May	3.694	3.825	3.726	3.926
2013	Jun	4.047	3.750	3.651	3.852
2013	Jul	3.690	3.535	3.442	3.631
2013	Aug	3.183	3.372	3.284	3.462
2013	Sep	2.903	3.399	3.312	3.489
2013	Oct	3.691	3.580	3.487	3.675
2013	Nov	4.260	3.713	3.611	3.818
2013	Dec	3.662	3.643	3.527	3.762
2014	Jan	3.695	3.824	3.707	3.945
2014	Feb	3.696	3.703	3.601	3.807
2014	Mar	3.923	3.753	3.657	3.852
2014	Apr	3.739	3.838	3.741	3.938
2014	May	3.658	3.861	3.763	3.962
2014	Jun	3.645	3.786	3.687	3.887
2014	Jul	3.570	3.569	3.476	3.664
2014	Aug	3.426	3.405	3.317	3.495
2014	Sep	3.412	3.432	3.345	3.521
2014	Oct	3.731	3.615	3.522	3.710
2014	Nov	3.513	3.749	3.647	3.855
2014	Dec	3.538	3.678	3.563	3.798
2015	Jan	4.270	3.862	3.744	3.984
2015	Feb	4.148	3.739	3.637	3.845
2015	Mar	3.722	3.790	3.693	3.890
2015	Apr	3.622	3.877	3.779	3.977
2015	May	3.320	3.900	3.800	4.002
2015	Jun	4.132	3.824	3.724	3.926
2015	Jul	4.100	3.605	3.511	3.701
2015	Aug	3.309	3.439	3.350	3.530
2015	Sep	3.733	3.467	3.379	3.557
2015	Oct	3.443	3.652	3.557	3.748
2015	Nov	3.757	3.787	3.683	3.895
2015	Dec	3.994	3.716	3.598	3.838
2016	Jan	3.511	3.901	3.780	4.026
2016	Feb	4.143	3.777	3.672	3.886
2016	Mar	3.533	3.829	3.729	3.932
2016	Apr	4.207	3.916	3.815	4.020
2016	May	3.365	3.940	3.837	4.046
2016	Jun	3.868	3.863	3.760	3.969
2016	Jul	3.601	3.642	3.545	3.742

2016	Aug	3.296	3.475	3.383	3.569
2016	Sep	3.768	3.503	3.411	3.597
2016	Oct	3.518	3.689	3.591	3.791
2016	Nov	3.788	3.827	3.716	3.940
2016	Dec	3.590	3.754	3.630	3.883
2017	Jan	3.835	3.942	3.814	4.073
2017	Feb	3.648	3.817	3.704	3.933
2017	Mar	3.896	3.869	3.760	3.981
2017	Apr	4.422	3.957	3.847	4.070
2017	May	4.168	3.981	3.868	4.097
2017	Jun	3.774	3.903	3.790	4.020
2017	Jul	3.863	3.680	3.572	3.791
2017	Aug	3.765	3.511	3.408	3.616
2017	Sep	3.637	3.539	3.436	3.645
2017	Oct	3.789	3.728	3.616	3.843
2017	Nov	3.530	3.866	3.741	3.996
2017	Dec	3.517	3.793	3.655	3.937
2018	Jan	3.985	3.983	3.838	4.133
2018	Feb	3.953	3.856	3.725	3.992
2018	Mar	4.019	3.909	3.779	4.043
2018	Apr	4.035	3.998	3.865	4.136
2018	May	3.911	4.022	3.885	4.164
2018	Jun	3.997	3.944	3.806	4.087
2018	Jul	3.564	3.718	3.586	3.855
2018	Aug	3.934	3.547	3.420	3.679
2018	Sep	3.270	3.576	3.447	3.710
2018	Oct	3.837	3.766	3.625	3.913
2018	Nov	3.760	4.082	3.911	4.261
2018	Dec	3.803	4.005	3.828	4.190
2019	Jan	4.039	4.205	4.017	4.401
2019	Feb	4.323	4.071	3.899	4.252
2019	Mar	4.929	4.127	3.957	4.304
2019	Apr	4.420	4.221	4.049	4.401
2019	May	4.606	4.246	4.072	4.428
2019	Jun	3.924	4.164	3.992	4.343
2019	Jul	3.587	3.925	3.763	4.094
2019	Aug	3.782	3.745	3.591	3.906
2019	Sep	3.736	3.775	3.620	3.937
2019	Oct	3.883	3.976	3.813	4.147
2019	Nov	3.813	4.124	3.952	4.304
2019	Dec	4.170	4.046	3.866	4.235

Table S2: Monthly observed and estimated stillbirth rates for Germany except Thuringia, from 1995 until 2019, estimated by negative binomial model in a Generalized Additive Model framework controlling for definition change and seasonality, estimated ‘overdispersion-theta’ is 1029.215 [16,17,33-50]
